# Hexaconazole-Induced Male Reproductive Toxicity Through ROS-Mediated Ferroptosis and Impaired Leydig Cell Steroidogenesis

**DOI:** 10.3390/ijms27146428

**Published:** 2026-07-20

**Authors:** Ran Lee, Hyeon Woo Sim, Won-Young Lee, Hyun-Jung Park

**Affiliations:** 1 Department of Livestock, Korea National University of Agriculture and Fisheries, Jeonju-si 54874, Jeonbuk, Republic of Korealeewy81@korea.kr (W.-Y.L.); 2 Department of Biotechnology, College of Biomedical & Health Science, Konkuk University, Chungju 27475, Chungbuk, Republic of Korea; 3 Department of Animal Biotechnology, College of Life Science, Sangji University, Wonju-si 26339, Gangwon-do, Republic of Korea

**Keywords:** hexaconazole, ferroptosis, ROS, steroidogenesis, Leydig cell

## Abstract

Triazole fungicides are ubiquitous environmental contaminants. However, their specific effects on the male reproductive system remain unclear. We investigated the toxicological effects of hexaconazole (HEX) on testicular function and elucidated its underlying molecular mechanisms. Although 8 weeks of HEX administration in mice did not affect body weight or gross testicular morphology, it significantly reduced sperm motility and circulating testosterone levels. Correspondingly, HEX exposure markedly downregulated germ cell (DDX4) and meiotic markers (SYCP3) as well as key steroidogenic related gene, including *3β-HSD*, *Cyp11a1*, and *Star*. HEX induced the excessive production of reactive oxygen species (ROS) and elicited molecular and biochemical features consistent with ferroptosis, an iron-dependent form of regulated cell death, predominantly in interstitial Leydig cells. This process is characterized by increased intracellular labile iron accumulation, enhanced lipid peroxidation (BODIPY fluorescence), and pronounced dysregulation of ferroptosis-associated regulators, including the upregulation of *Tfrc*, *Slc11a2*, and *Acsl4*, concomitant with substantial depletion of *Gpx4*, an antioxidant related gene. Consistent with in vivo findings, in vitro experiments using primary Leydig cells demonstrated that HEX-induced oxidative stress directly compromised cell viability and steroidogenic function. Thus, Leydig cell ferroptosis is associated with underlying HEX-induced reproductive toxicity, linking endocrine disruption to impaired spermatogenesis. Furthermore, we identified the ferroptosis-mediated antioxidant defense system as a potential molecular target for mitigating reproductive risks associated with triazole fungicide exposure.

## 1. Introduction

Hexaconazole (HEX) is a systemic triazole fungicide belonging to the conazole class of azole fungicides; it is widely used in agriculture to control fungal diseases in crops such as rice, grapes, and tomatoes [1,2]. As a sterol biosynthesis inhibitor, HEX targets the cytochrome P450-dependent 14α-demethylase (CYP51) in fungi to inhibit ergosterol biosynthesis, thereby disrupting the integrity of the fungal cell membrane and ultimately killing the fungus. Despite its effectiveness in crop protection, increasing concerns have been raised regarding the potential adverse effects of HEX on non-target organisms, including mammals [3,4]. HEX residues have been widely detected in environmental matrices such as soil, surface water, and agricultural products, suggesting their persistence and the potential for chronic exposure [5]. Due to its frequent use and relatively slow degradation rate, HEX can repeatedly enter and persist in the agricultural environment. Typically, HEX is applied 3–4 times at intervals of 7–14 days per crop growth cycle [6]. According to environmental monitoring studies, HEX concentrations ranging from ng/kg to mg/kg have been reported in agricultural soils, with residual concentrations of approximately 0.78–1.66 mg/kg still detected 90 days after application [6,7]. Furthermore, accumulation of HEX and its isomers has been observed in soil microorganisms, which may cause oxidative stress and biological disruption [6]. These findings suggest that continuous exposure to HEX may pose a potential risk to non-target organisms.

According to toxicological studies, exposure to HEX can cause various adverse effects, including endocrine disruption, immunotoxicity, neurotoxicity, and metabolic abnormalities [8,9]. In zebrafish larvae, HEX and other triazole-based fungicides were found to alter gene expression related to the hypothalamic-pituitary-thyroid (HPT) axis, leading to reduced thyroid hormone levels and endocrine disruption [8]. Furthermore, HEX and related triazole-based fungicides have been reported to cause metabolic disorders related to energy metabolism, lipid metabolism, and amino acid pathways [9]. In terms of acute toxicity, HEX is classified as a low- to moderately toxic compound in mammals, and the reported oral LD50 values indicate relatively limited acute toxicity following exposure [10]. However, since repeated or chronic exposure to HEX can cause biological disruption through endocrine and metabolic mechanisms, further investigation into the long-term toxicological consequences of HEX exposure is warranted. Previous studies have suggested that triazole fungicides, including HEX, adversely affect reproductive function through endocrine-disrupting mechanisms. However, the molecular mechanisms underlying HEX-induced reproductive dysfunction have not yet been fully elucidated. Wang et al. reported that triazole-based fungicides exhibit estrogen-related endocrine-disrupting activity and may interfere with estrogen signaling pathways. HEX alters estrogen receptor alpha (ERα)-related activity and genes involved in steroidogenesis (steroid biosynthesis acute regulatory protein (StAR), cytochrome P450 family 11 (CYP11A1), 3β-hydroxysteroid dehydrogenase type 2 (3β-HSD2), 17α-hydroxylase/C17,20-lyase (CYP17), and cytochrome P450 family 19 subfamily A member 1 (CYP19)) [11]. Although this study did not directly investigate ERα activation, these previous findings suggest that HEX may influence reproductive endocrine pathways and support the need for further research into steroid production dysfunction in Leydig cells.

Since Leydig cells play a crucial role in testosterone synthesis and male reproductive function, it is essential to investigate the effects of HEX on steroid production in Leydig cells. Although not an in vivo experiment, Roelofs et al. used an in vitro model to study the effects of conazole-class fungicides on testosterone secretion and androgen receptor activation in MA-10 Leydig cells. Ciproconazole (CYPRO), flusilazole (FLUS), HEX, prochloraz (PRO), tebuconazole (TEBU), and triticonazole (TRIT) significantly suppressed testosterone secretion; in particular, FLUS and TEBU exhibited strong, dose-dependent inhibitory effects. All conazole compounds except FLUS also inhibited androgen receptor activation. Furthermore, when exposed to the highest concentration (100 μM), MA-10 cells treated with FLUS, HEX, PRO, and TEBU exhibited increased intracellular ROS generation compared to control cells, suggesting that oxidative stress may contribute to conazole-induced Leydig cell dysfunction [12].

In this study, we investigated the reproductive toxicity of HEX and examined whether oxidative stress-associated ferroptotic signaling contributes to Leydig cell dysfunction and impaired steroidogenesis, thereby providing mechanistic insight into HEX-induced male reproductive toxicity.

## 2. Results

### 2.1. Impact of HEX Treatment on Murine Testes and Germ Cells

**Figure 1:**
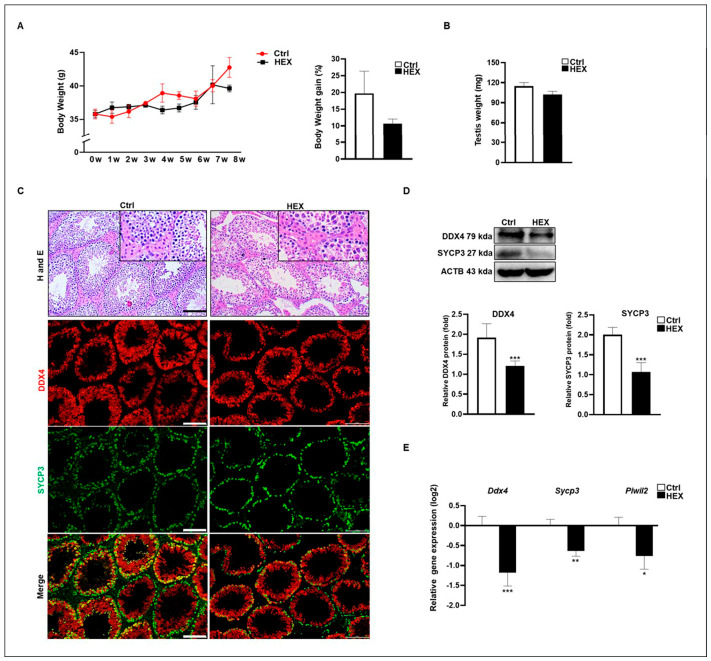
HEX exposure affects testicular germ cells. (**A**) Body weight from each experimental group. Data are presented as mean ± SD (*n* = 10). (**B**) Testis weight was measured (*n* = 10). (**C**) Hematoxylin and eosin staining of the testis from each group. Immunostaining of DDX4 and SYCP3 in testes. Scale bar = 100 μm (*n* = 3). (**D**) Representative immunoblot images showing the protein expression of DDX4, SYCP3, and ACTB in testes from each group. The graph is presented as mean ± SD (*n* = 3) with fold values. *** *p* < 0.001. (**E**) Gene expression of Ddx4, Sycp3, and Pwil2 from each experiment group. The graph shows mean ± SD (*n* = 5) with a log2 scale. * *p* < 0.05, ** *p* < 0.01, and *** *p* < 0.001.

Eight weeks of HEX administration did not significantly affect body weight or testis weight in mice (Figure 1A,B). Histological examination of H&E-stained testicular sections revealed no overt histopathological alterations in the seminiferous tubules or interstitial compartment following HEX exposure. Immunohistochemical analysis demonstrated the presence of DDX4-positive germ cells and SYCP3-positive meiotic cells in both experimental groups. No obvious differences in the distribution of DDX4- or SYCP3-positive cells were observed between the control and HEX-treated groups (Figure 1C). Western blot analysis demonstrated that DDX4 and SYCP3 protein expression was significantly decreased in the testes of HEX-treated mice compared with controls (Figure 1D). Consistent with these findings, quantitative PCR analysis revealed significant downregulation of *Ddx4*, *Sycp3*, and *Piwil2* mRNA expression following HEX exposure (Figure 1E).

### 2.2. Effect of HEX on Steroidogenesis and Sperm Parameters in Mouse Testes

**Figure 2:**
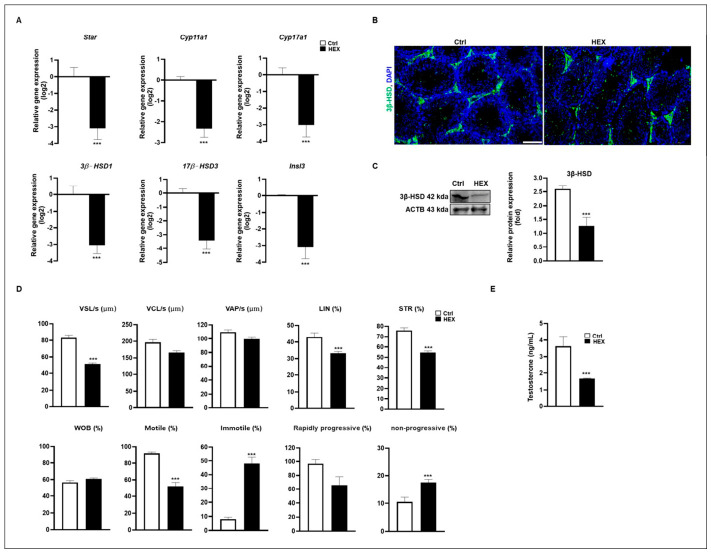
HEX exposure affects steroidogenesis and sperm parameters in the testes. (**A**) Relative mRNA expression of *Star*, *Cyp11a1*, *Cyp17a1*, *3β-HSD1*, *17β-HSD3*, and *Insl3* in the testes from each group. Data are presented as mean ± SD on a log2 scale (*n* = 5), *** *p* < 0.001. (**B**) Immunohistochemical staining of 3β-HSD in the testes from HEX-injected and control mice. Scale bar = 100 µm. (**C**) Representative immunoblot images showing the protein expression of 3β-HSD and ACTB, along with the quantitative graph (mean ± SD, *n* = 3), *** *p* < 0.001. (**D**) Sperm motility and related parameters were analyzed, including velocity straight line (VSL), velocity curvilinear (VCL), velocity average path (VAP), linearity (LIN), straightness (STR), wobble (WOB), motile (%), immotile (%), rapidly progressive (%), and non-progressive (%). *** *p* < 0.001 (*n* = 5). (**E**) Testosterone levels were detected in each experiment group. Graph is represented as mean ± SD (*n* = 5). *** *p* < 0.001.

Having demonstrated that HEX impaired germ cell-associated markers, we next investigated whether HEX affected testicular steroidogenesis. Next, we investigated whether HEX influenced testicular steroidogenesis. The expression of steroidogenesis-related genes, including *Star*, *Cyp11a1*, *Cyp17a1*, *3β-HSD1*, and *17β-HSD3*, was significantly downregulated following HEX exposure. Similarly, the expression of *Insl3*, a Leydig cell-specific marker, was significantly reduced in the HEX-treated group (Figure 2A). To determine whether these transcriptional changes were reflected at the protein level, 3β-HSD expression was evaluated by immunohistochemistry and Western blotting. Immunohistochemical analysis revealed markedly reduced 3β-HSD immunoreactivity in the interstitial compartment of HEX-treated testes compared with controls (Figure 2B). Consistent with these findings, Western blot analysis confirmed a significant reduction in 3β-HSD protein expression in the HEX-treated group (Figure 2C).

Given that abnormal steroidogenesis has been linked to reduced sperm function [13,14], we next analyzed the sperm parameters in each group following these references. The parameters examined included velocity straight line (VSL), velocity curvilinear (VCL), velocity average path (VAP), linearity (LIN), straightness (STR), wobble (WOB), motility, immotility, rapid progression, and non-progression. Compared to the control group, HEX exposure significantly reduced VSL, LIN, STR, motility, and rapid progression, whereas immotility and non-progression were significantly increased (Figure 2D). In addition, the concentration of testosterone in the blood serum was measured, and the results showed a significant decrease in HEX-exposed mice compared to control mice (Figure 2E). Altogether, HEX exposure partially suppressed normal steroid genesis and sperm production in the testes.

### 2.3. HEX Triggers ROS Generation and Induces Ferroptosis in the Testes

**Figure 3:**
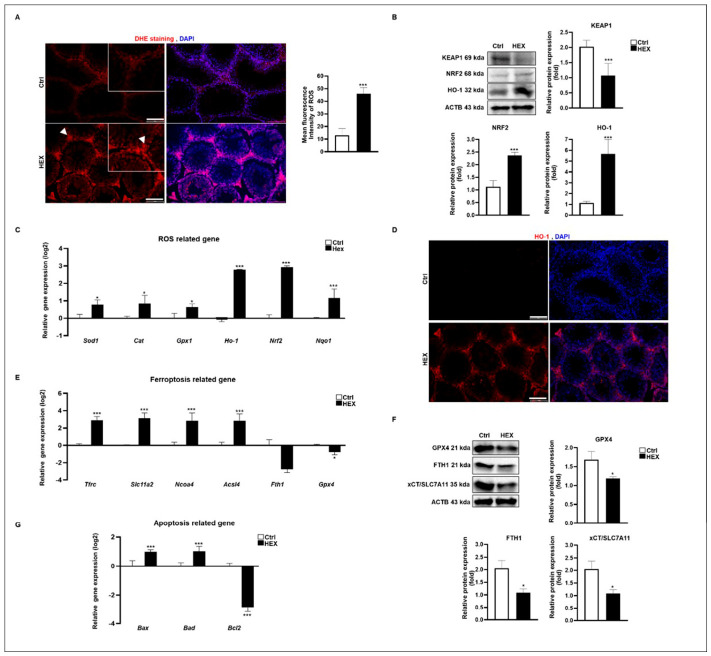
HEX induces ROS and ferroptosis in testes. (**A**) Dihydroethidium (DHE) staining in each sample and DAPI-stained nucleus. Scale bar = 50 μm. The graph represents the mean fluorescence intensity of ROS. *** *p* < 0.001 (*n* = 3). White arrows indicate the testicular interstitium. (**B**) Expression of Keap1, Nrf2, HO-1, and ACTB proteins in the testis of each group. *** *p* < 0.001 (*n* = 3). (**C**) Gene expression of *Sod1*, *Cat*, *Gpx1*, *Ho-1*, *Nrf2*, and *Nqo1* in the testis from each group. The graph shows the mean ± SD (*n* = 5) with a log2 scale. * *p* < 0.05 and *** *p* < 0.001. (**D**) Immunostaining of HO-1 in each testis sample and DAPI-stained nucleus. Scale bar = 100 μm (*n* = 3). (**E**) Gene expression of *Tfrc*, *Slc11a2*, *Ncoa4*, *Acsl4*, *Fth1*, and *Gpx4* in the testis from each group. The graph shows the mean ± SD with a log2 scale (*n* = 5). * *p* < 0.05 and *** *p* < 0.001. (**F**) The protein expression of GPX4, FTH1, xCT/SLC7A11, and ACTB. The graph represents the mean ± SD, and relative protein expression was normalized to that of ACTB. * *p* < 0.05 (*n* = 3). (**G**) Gene expression of *Bax*, *Bad*, and *Bcl2* in the testis from each group. The graph shows the mean ± SD with a log2 scale (*n* = 5). *** *p* < 0.001.

Based on the observed impairment of germ cell-associated markers and steroidogenic function following HEX exposure, we next investigated the underlying molecular mechanisms. Because triazole fungicides have been reported to induce oxidative stress in multiple tissues [15,16], we first evaluated ROS generation in the testes by DHE staining. DHE fluorescence intensity was markedly increased in the testes of HEX-treated mice compared with controls, with the strongest signal detected in the testicular interstitium (Figure 3A, white arrow). Quantitative analysis confirmed a significant increase in DHE fluorescence intensity following HEX exposure. To further characterize the oxidative stress response, we analyzed the expression of antioxidant defense-related proteins. Western blot analysis demonstrated increased expression of NRF2 and HO-1, accompanied by reduced *KEAP1* expression in the testes of HEX-treated mice (Figure 3B). Consistent with these findings, Sod1, *Cat*, *Gpx1*, *Ho-1*, *Nrf2*, and *Nqo1* mRNA expression levels were significantly upregulated (Figure 3C), and HO-1 immunoreactivity was markedly enhanced in the HEX-treated testes (Figure 3D). Previous studies have demonstrated that oxidative stress induces ferroptosis in cells [17,18]. Based on these reports, we examined whether ferroptosis occurs in the testes following HEX treatment. Ferroptosis-related genes, including *TFR1* (*Tfrc*), *Slc11a2* (Solute carrier family 11 member 2/Divalent metal transporter 1), *Ncoa4* (nuclear receptor coactivator 4), *Fth1* (ferritin heavy chain 1), and *Gpx4* (glutathione peroxidase 4), were detected in each sample. Transcription levels of *Tfrc*, *Slc11a2*, *Ncoa4*, and *Acsl4* (acyl-coA synthetase long chain family member 4) were significantly increased in HEX-exposed mouse testes, whereas those of *Fth1* and *Gpx4* were decreased (Figure 3E). To verify the gene expression data, protein levels of GPX4, FTH1, and xCT/SLC7A11 were measured in each sample. The results showed that the protein levels of DMT1 increased, and those of GPX4, FTH1, and xCT/SLC7A11 decreased in the testes of HEX-exposed mice (Figure 3F). In addition, ferroptosis-related alterations were accompanied by changes in apoptosis-related markers. The expression levels of *Bax* (BCL2-associated X protein) and *Bad* (BCL2-associated agonist of cell death) were increased at both the mRNA and protein levels, whereas *Bcl2* (B-cell lymphoma 2) expression was reduced in HEX-exposed groups (Figure 3G).

### 2.4. Isolation of Primary Leydig Cells and Effect of HEX on Steroidogenesis in Primary Leydig Cells

**Figure 4:**
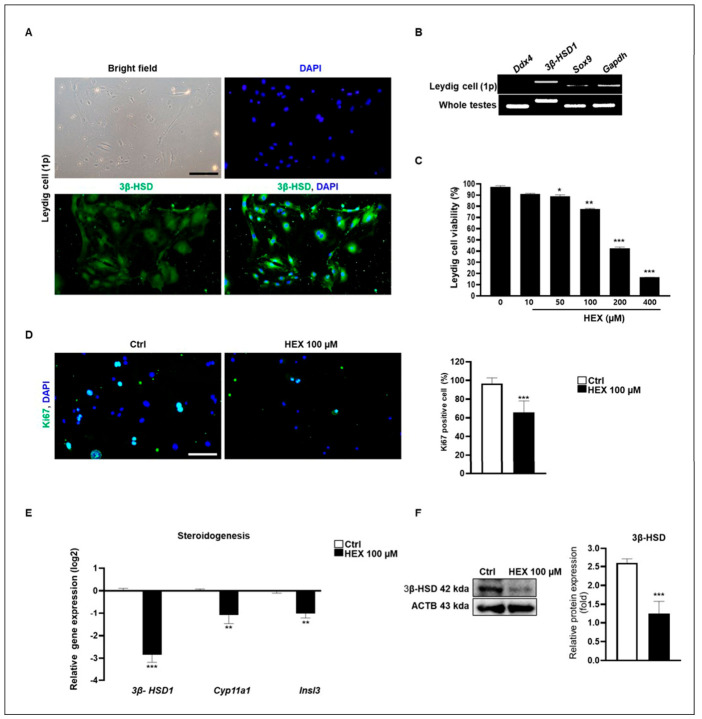
Isolation of primary Leydig cells and the impact of HEX on steroidogenesis. (**A**) Immunostaining of 3*β*-HSD and DAPI in primary Leydig cells (passage 1). Scale bar = 100 µm (*n* = 3). (**B**) Gene expression of *Ddx4*, *3β-HSD1*, *Sox9*, and *Gapdh* in primary Leydig cells and whole testes (*n* = 5). (**C**) Relative cell viability (%) of primary Leydig cells after 24 h with 0–400 μM HEX treatment. * *p* < 0.05, ** *p* < 0.01, and *** *p* < 0.001. (**D**) Immunostaining of Ki67 in each sample. Graph shows the mean ± SD (%, *n* = 3). *** *p* < 0.001. (**E**) Gene expression of *3β-HSD1*, *Cyp11a1*, and *Ins3* in each sample by qPCR. Data are presented as mean ± SD with log2 values. ** *p* < 0.01 and *** *p* < 0.001 (*n* = 5). (**F**) Protein levels of 3*β*-HSD with densitometric analysis normalized to the level of ACTB. Values represent the mean ± SD from three independent experiments (*n* = 3). *** *p* < 0.001.

DHE-positive fluorescence was predominantly localized to the interstitial compartment of the testis, suggesting that Leydig cells may represent a major target of HEX-induced oxidative stress (Figure 3A). To directly investigate the effects of HEX on Leydig cells, primary Leydig cells were isolated from mouse testes as previously described by Liang et al. [19]. Immunostaining for 3β-HSD demonstrated that more than 90% of the isolated cells were positive for this Leydig cell marker, indicating high cell purity (Figure 4A). Consistent with these findings, RT-PCR analysis revealed clear expression of the Leydig cell marker *3β-HSD1*, whereas *Ddx4* (a germ cell marker) was undetectable and Sox9 (a Sertoli cell marker) was detected only at trace levels (Figure 4B), confirming minimal contamination by non-Leydig cell populations. Cell viability analysis demonstrated that HEX significantly reduced the viability of primary Leydig cells in a concentration-dependent manner over the range of 50–400 μM (Figure 4C). The calculated IC_50_ value was 169.7 μM. Based on these findings, 100 μM HEX was selected for all subsequent in vitro experiments. Following exposure to 100 μM HEX for 24 h, the proportion of Ki67-positive cells was significantly reduced compared with the control group, indicating impaired proliferative capacity (Figure 4D). The expression of steroidogenesis-related genes, *3β-HSD1* and *Cyp11a1*, and *Ins3* is distinctly downregulated in HEX-exposed Leydig cells (Figure 4E). Following exposure to 100 μM HEX for 24 h, the proportion of Ki67-positive cells was significantly reduced compared with the control group, indicating impaired proliferative capacity (Figure 4D).

### 2.5. ROS Production and Ferroptosis Were Triggered by HEX Treatment

**Figure 5:**
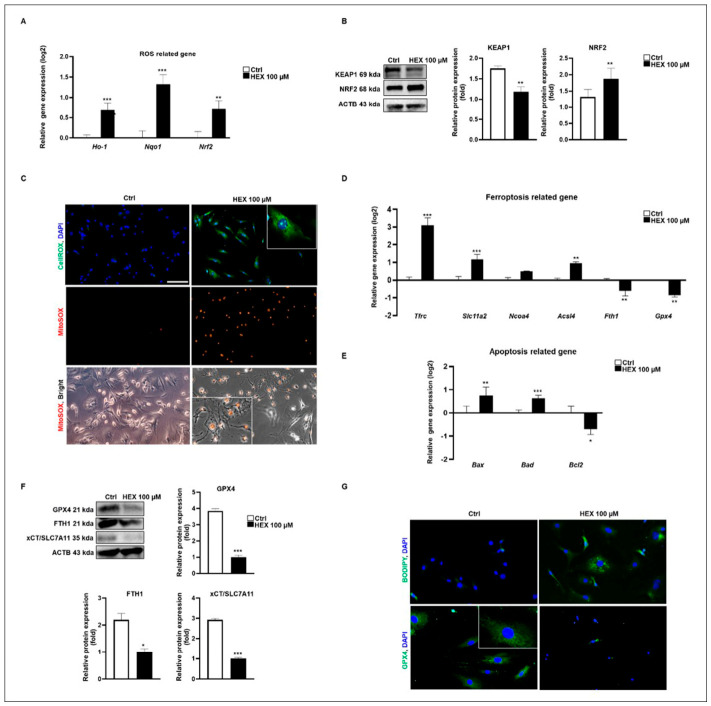
HEX treatment induced ROS generation and ferroptosis. (**A**) Gene expression of *Ho-1*, *Nqo-1*, and *Nrf2* in each group, as determined by qPCR. Data are represented as mean ± SD (*n* = 5). ** *p* < 0.01 and *** *p* < 0.001 compared to controls. (**B**) Protein expression of Keap1 and Nrf2 in each group. Relative protein expression to ACTB, which is the loading control, is shown as a graph with fold values and mean ± SD (*n* = 3). ** *p* < 0.01 compared to controls. (**C**) CellROX and MitoSOX staining of Leydig cells from each experimental group. Scale bar = 50 μm (*n* = 3). (**D**) Gene expression of *Tfrc*, *Slc11a2*, *Ncoa4*, *Acsl4*, *Fth1*, and *Gpx4* in each sample. Data are shown as mean ± SD with log2 values. ** *p* < 0.01 and *** *p* < 0.001 indicate significant differences compared to the control group (*n* = 5). (**E**) Gene expression of *Bax*, *Bad*, and *Bcl2* in the testis from each group. The graph shows the mean ± SD with a log2 scale (*n* = 5). * *p* < 0.05, ** *p* < 0.001, and *** *p* < 0.0001. (**F**) Protein levels of GPX4, FTH1, and xCT/SLC7A11 with densitometric analysis normalized to the level of ACTB. Values represent the mean ± SD from three independent experiments (*n* = 3). * *p* < 0.05, and *** *p* < 0.0001. (**G**) Immunostaining of GPX4 in each sample. BODIPY was also used to stain cells from each experiment group. Scale bar = 50 μm (*n* = 3).

The in vivo findings presented in Figure 3 demonstrated enhanced oxidative stress together with molecular alterations associated with ferroptosis in the tests following HEX exposure. To determine whether these responses also occurred at the cellular level, primary Leydig cells were analyzed. Consistent with the in vivo findings, quantitative PCR analysis revealed significant upregulation of the oxidative stress-responsive genes *Ho-1*, *Nqo1*, and *Nrf2* in HEX-treated Leydig cells compared with controls (Figure 5A). At the protein level, NRF2 expression was increased, whereas KEAP1 expression was decreased following treatment with 100 μM HEX (Figure 5B), indicating activation of the NRF2-mediated antioxidant response. Fluorescence-based ROS assays further supported these findings. CellROX fluorescence intensity was significantly increased in HEX-treated Leydig cells compared with controls. In addition, MitoSOX staining demonstrated increased mitochondrial superoxide production following HEX exposure (Figure 5C). We next investigated whether HEX induced molecular alterations consistent with ferroptosis in primary Leydig cells. Quantitative PCR analysis demonstrated significant upregulation of *Tfrc*, *Slc11a2*, *Ncoa4*, and *Acsl4*, whereas *Fth1* and *Gpx4* expression was significantly downregulated following HEX treatment (Figure 5D). Consistent with these findings, Western blot analysis revealed decreased expression of GPX4, FTH1, and xCT/SLC7A11 proteins in HEX-treated Leydig cells (Figure 5F). Immunofluorescence staining further confirmed reduced GPX4 immunoreactivity following HEX exposure (Figure 5G). In addition, BODIPY staining demonstrated a significant increase in lipid peroxidation in HEX-treated Leydig cells compared with controls (Figure 5G). Alterations in ferroptosis-associated markers were accompanied by increased expression of the pro-apoptotic markers *Bax* and *Bad* and decreased expression of the anti-apoptotic marker *Bcl2* (Figure 5E). Collectively, these findings indicate that HEX induces oxidative stress and promotes molecular and biochemical alterations consistent with ferroptosis in primary Leydig cells, in agreement with the observations obtained in vivo.

## 3. Discussion

Triazole fungicides are extensively used in agriculture and are frequently detected in the environment and in living organisms. However, their effects on animal development and physiology remain poorly understood. In the present study, we focused on the impact of HEX on testicular functions and explored the molecular mechanisms underlying its toxicity in testicular cells, particularly Leydig cells. This study is noteworthy, as it represents the first investigation of the effects of HEX on male reproductive function and toxicity in rodents. Our study demonstrated that although HEX exposure in mice did not affect body or testis weight, it reduced the expression of germ cells and steroidogenesis markers, lowered testosterone, and impaired sperm motility. HEX induced ROS generation and molecular alterations consistent with ferroptosis in the testes, as evidenced by oxidative stress markers, altered antioxidant responses, and ferroptosis-associated gene and protein expression. Similar findings were observed in isolated primary Leydig cells, which exhibited reduced viability, impaired steroidogenesis, increased ROS production, molecular features consistent with ferroptosis, and enhanced apoptotic signaling.

In addition to direct effects on germ cells, steroid-producing dysfunction in Leydig cells may be a potential underlying mechanism of HEX-induced reproductive toxicity [20]. Testosterone biosynthesis in Leydig cells begins when cholesterol is transported to mitochondria via acute regulatory protein for steroid production (StAR) and converted to pregnenolone by the cholesterol side-chain cleavage enzyme, CYP11A1. Subsequently, pregnenolone is converted to testosterone through a series of enzymatic reactions involving CYP17A1 and 17β-hydroxysteroid dehydrogenase (17B-HSD), and luteinizing hormone (LH)-mediated signaling regulates the expression and activity of these steroid-producing enzymes [21].

Given that triazole fungicides can disrupt cytochrome P450-dependent pathways, HEX may interact with the heme-containing catalytic domains of CYP enzymes to interfere with androgen biosynthesis, potentially damaging the Leydig cell-spermiogenesis axis [22]. Therefore, changes in steroid-producing signaling may contribute to the reproductive dysfunction and reduction in germ cell-related indicators observed after HEX exposure [20].

Consistent with these potential mechanisms, previous studies have also revealed that other azole fungicides exhibit endocrine-disrupting activity. Svanholm et al. investigated the effects of the azole fungicides propiconazole (PROP) and imazalil (IMZ) on young Xenopus tropicalis [23]. Exposure to PROP resulted in altered expression of retinoic acid-related genes in the testes and ovaries without causing distinct histological changes, whereas exposure to IMZ inhibited spermatogenesis, increased the number of dark-colored spermatogonial stem cells, and affected the expression of genes involved in germ cell formation and sex steroid pathways [23]. Additionally, PROP and IMZ have been reported to inhibit the activity of CYP1, CYP17, and CYP19 (aromatases) and antagonize androgen receptor (AR) signaling, suggesting that azole antimicrobial agents may affect reproductive function through various endocrine-related mechanisms [24,25,26,27]. Our results also demonstrated that HEX reduced the expression of germ cell markers such as DDX4 and SYCP3. SYCP3 is a meiotic marker of germ cells, and reduced expression of meiotic markers indicates suppression of critical steps required for the progression of spermatogenesis. Previous studies have shown that steroidogenic cells are particularly susceptible to oxidative damage because steroid biosynthesis is tightly coupled to mitochondrial function, resulting in increased reactive oxygen species generation during high metabolic activity [28,29]. Although decreased expression of germ cell markers (DDX4 and SYCP3) and impaired sperm motility indicate that germ cells are affected by HEX exposure, the localization of ROS signals suggests that Leydig cells represent the primary site of initial damage. Because testosterone produced by Leydig cells is essential for maintaining spermatogenesis, it is highly plausible that Leydig cell dysfunction leads to secondary impairment of germ cell development. Recently, the endocrine-disrupting potential of HEX has also been reported, and this study showed that HEX stably binds to sex hormone-binding globulin (SHBG) with molecular dynamics similar to that of natural ligands, suggesting that it may disrupt steroid hormone activity and pose an endocrine-disrupting risk during agricultural use [30]. Our results also demonstrated dramatically reduced expression of steroidogenesis-related genes in the testes after HEX exposure and decreased serum testosterone concentration. Such abnormal steroidogenic processes are likely to have resulted in reduced sperm parameters, as observed in our results. A revalidation experiment using primary Leydig cells isolated from mouse testes demonstrated the negative effect of HEX on steroidogenesis.

Several studies have demonstrated that HEX enhances cellular damage in mammals. HEX impairs cognition in rats by inducing oxidative stress and disrupting neurotransmission and biological rhythm [31]. Another study reported that HEX disrupts lipid homeostasis in rats by inducing oxidative stress, fat accumulation, and histopathological damage under subchronic exposure. Lipidomics analyses revealed activation of the mTOR-PPAR-γ/SREBP1 signaling pathway [32]. Although the target organs and cell types differed, both studies reported that HEX induced ROS generation. Consistent with these findings, our results demonstrate ROS production in the testes. The KEAP1–NRF2 signaling pathway plays a key role in cellular antioxidant defense. Under conditions of oxidative stress, NRF2 detaches from KEAP1 and translocates to the nucleus, where it induces the expression of antioxidant genes such as Ho-1 and Nqo1 [33]. This study shows that increased NRF2 expression and increased downstream target gene expression in response to HEX exposure activate this pathway. However, this compensatory antioxidant response appears insufficient to offset the excessive accumulation of reactive oxygen species, ultimately leading to oxidative damage. These effects were particularly evident in Leydig cells. Although HEX exposure led to downregulation of both germ cell and steroidogenic markers in the testes, DHE staining revealed that ROS signals were predominantly localized in the interstitial part of the testes. This spatial pattern indicates that Leydig cells are a primary target of HEX-induced oxidative stress. To further investigate this possibility, in vitro experiments were performed using isolated primary Leydig cells.

In experiments using Leydig cells, the number of passages was limited to three, and we confirmed that steroidogenesis-related genes were endogenously expressed, and HEX suppressed the expression of these genes in primary Leydig cells. Our results in Leydig cells are supported by evidence from the literature. Abdi et al. reported that continuous exposure to HEX disrupts steroid hormone synthesis. In particular, molecular interactions between HEX and key enzymes are responsible for the progression of steroid hormone synthesis [34]. Ferroptosis has recently been discovered as an iron-dependent cell death pathway, characterized by iron accumulation and lipid peroxidation. This occurs when ferroptosis-inducing factors reduce antioxidant capacity, particularly by affecting glutathione peroxidase, leading to ROS build-up and oxidative cell death. Ferroptosis has been implicated in several diseases, such as cancer, neurological disorders, ischemia/reperfusion injury, kidney damage, and blood disorders. Understanding and regulating ferroptosis is a growing research focus for disease treatment [35]. Our results demonstrated dysregulated expression of key molecular regulators associated with ferroptosis in both the testes and primary Leydig cells following HEX exposure. Tfrc mediates iron uptake into cells by binding to the transferrin–iron complex, and increased Tfrc levels promote intracellular iron accumulation, fueling ferroptosis. In addition, ferroptosis involves a complex interplay between genes and proteins that regulate intracellular iron metabolism, lipid peroxidation, and antioxidant defense. Among these, DMT1, NCOA4, ACSL4, FTH1, and GPX4 play critical roles in controlling ferroptosis. DMT1 facilitates the import of ferrous iron (Fe^2+^) into cells and organelles. Elevated DMT1 expression increases the intracellular iron availability, thereby enhancing the susceptibility of cells to ferroptosis. Simultaneously, NCOA4 mediates ferritinophagy, a selective autophagic process that degrades ferritin and releases stored iron into the cytosol. This process increases the labile iron pool and promotes ferroptosis. ACSL4 catalyzes the synthesis of polyunsaturated fatty acid-containing phospholipids, which serve as substrates for lipid peroxidation, a key step in ferroptosis. In contrast, FTH1 plays a protective role by storing iron in a non-toxic form within ferritin complexes. Reduced FTH1 expression limits iron storage capacity, leading to the accumulation of free iron and an increased risk of ferroptosis cell death. GPX4 acts as a crucial antioxidant enzyme that detoxifies lipid hydroperoxides, thereby preventing lipid peroxidation and maintaining membrane integrity. The inhibition or depletion of GPX4 directly triggers ferroptosis, underscoring its essential role as a defense mechanism against oxidative cell death. Dysregulation of these ferroptosis-related molecules disrupts iron homeostasis and antioxidant balance, ultimately driving oxidative damage and cell death [36,37]. Generally, ROS are involved in several types of cell death or damage, including apoptosis, necrosis, autophagy, and ferroptosis [38]. Ferroptosis is an iron-dependent cell death process characterized by the accumulation of lipid peroxides. Ferroptosis occurs only when ROS specifically cause iron-dependent lipid peroxidation and GPX4 fails to detoxify lipid peroxides [39]. BODIPY staining is widely used to assess lipid peroxidation, a characteristic biochemical feature associated with ferroptosis. Our results demonstrated strong BODIPY signals in HEX-exposed primary Leydig cells; moreover, the number of GPX4-positive cells was markedly reduced by HEX treatment (visual observation). These findings support the involvement of ferroptosis-associated signaling in HEX-induced cellular damage.

Regarding the potential effects of HEX on the Leydig cell-spermiogenesis axis, we observed that 8 weeks of HEX administration did not cause significant changes in body weight or testicular weight, and the overall histological structure of the testes was relatively well preserved. According to previous toxicological studies, mice exposed to high concentrations of hexaconazole (up to 225 mg/kg/day) exhibited weight loss and liver toxicity, whereas at lower concentrations, a no-observed-adverse-effect level (NOAEL) of 3.75 mg/kg/day was identified after 29 days of dietary administration [40]. These results suggest that exposure to HEX at the test doses did not cause distinct changes in the general toxicity indicators evaluated in this study or induce severe testicular structural damage [41]. However, the significant downregulation of DDX4 and SYCP3 suggests that HEX may impair spermiogenesis at a molecular level, particularly affecting germ cell maintenance and meiotic progression. Further studies evaluating additional systemic toxicity indicators are necessary to comprehensively elucidate the broad toxicological effects of HEX exposure.

The decline in germ cell markers warrants careful interpretation of whether this is a direct toxic effect on the germinal epithelium or a secondary consequence of interstitial dysfunction [42,43]. In our study, the dramatic suppression of Insl3 and key steroidogenic enzymes (3β-HSD1 and Cyp11a1) suggests that the Leydig cells in the interstitial part of the testes are highly sensitive targets of HEX. Because Leydig cell-derived testosterone is indispensable for maintaining the specialized microenvironment required for meiosis and sperm maturation, it is highly plausible that HEX-induced Leydig cell failure contributes to the observed impairment of spermatogenesis. However, we cannot rule out the possibility that HEX directly targets germ cells or disrupts Sertoli cell support. The strong ROS signals and ferroptosis-associated molecular changes observed within the interstitial compartment (as demonstrated by DHE and GPX4 staining) support the hypothesis that Leydig cell dysfunction is an important contributor to the observed reproductive impairment. Several limitations of the present study should be acknowledged. First, although our data consistently demonstrated oxidative stress and dysregulation of multiple ferroptosis-associated markers, definitive confirmation of ferroptosis would require pharmacological rescue experiments using established ferroptosis inhibitors such as Ferrostatin-1 or Liproxstatin-1. Future studies incorporating these inhibitors will be important to establish the causal contribution of ferroptosis to HEX-induced Leydig cell dysfunction. Second, the present study evaluated only a single in vivo dose of HEX. Additional dose–response studies are warranted to further characterize the toxicological relevance and dose-dependent effects of HEX on male reproductive function.

## 4. Materials and Methods

### 4.1. Animals and Treatment

Seven-week-old C57BL/6 male mice (*n* = 20) were obtained from Daehan Biolink Co., Ltd. (Daejeon, Republic of Korea) and allowed to acclimate for 1 week before the experiments. Animals were maintained under controlled conditions with a 12 h light/12 h dark cycle at 21 ± 1 °C. All experimental procedures were approved by the Institutional Animal Care and Use Committee of Sangji University (Registration No. #2023-10 approved on 25 October 2023, and Registration No. #2025-3, approved on 28 July 2025). Hexaconazole (HEX; Sigma-Aldrich, St. Louis, MO, USA, #34348) was prepared by dissolving in a small volume of dimethyl sulfoxide (Sigma-Aldrich, St. Louis, MO, USA; #D8418) and subsequently diluting with phosphate-buffered saline (PBS; SPL Life Sciences Co., Ltd., Pocheon, Republic of Korea) to the required concentrations. Mice were randomly assigned to two groups of 10 animals each and treated daily by oral administration for 8 weeks: Group 1 (*n* = 10) received PBS, whereas Group 2 (*n* = 10) was administered 150 mg/kg HEX. The dosage of HEX was selected according to previously published studies [44]. The body weight was recorded weekly throughout the experimental period. At the end of the 8-week treatment period, the mice were euthanized, and the testes were excised to measure organ weight. Serum samples were collected for testosterone assays, and testicular tissue was either fixed in 4% formaldehyde for histological evaluation or stored at −80 °C for protein and RNA extraction.

### 4.2. Isolation of Primary Leydig Cells and Culture

Seven-week-old C57/BL6 male mice were sourced from Daehan Biolink and acclimatized for a week. The method of isolating primary Leydig cells and culturing has been described previously [19]. Briefly, mice were euthanized following institutional ethical guidelines and aseptically removed. Tunica albuginea was carefully removed, and the testicular tissue was minced into fragments using sterile scissors. The tissue was digested with 10 mg/mL Collagenase type IV (Gibco™, Thermo Fisher Scientific, Waltham, MA, USA; #17104019) solution at 37 °C for 20 min with gentle agitation. Following enzymatic digestion, the cell suspension was passed through a 40 μm cell strainer (Corning; Glendale, AZ, USA) to remove tissue debris. The filtrate was centrifuged at 250 *g* for 10 min. After centrifugation, the cell pellet was resuspended in 2 mL of complete DMEM (SPL Life Sciences Co., Ltd., Pocheon, Republic of Korea) supplemented with 10% heat-inactivated FBS (Gibco™, Thermo Fisher Scientific, Waltham, MA, USA) and 1% penicillin–streptomycin (SPL Life Sciences Co., Ltd., Pocheon, Republic of Korea). The cell suspension was evenly seeded into two 100 mm culture dishes, each containing 5 mL of complete medium; the dishes were incubated in a 5% CO_2_ incubator at 37 °C for 1 h. After 1 h, the non-adherent cells were removed, and 6 mL of fresh complete medium was added to each dish and cultured for 24 h. After 24 h, the medium was discarded, and the adherent cells were treated with 4 mL of KCl hypotonic solution for 5 min to further eliminate myoid cells, followed by three washes with PBS. Next, 6 mL of fresh complete medium was added per dish, and culturing continued for 48 h. After 48 h, LCs were harvested for further experiments. The purity of isolated Leydig cells was verified by HSD3B staining, and preparations with >85% HSD3B-positive cells were used for subsequent experiments. All experiments were performed using primary Leydig cells at passages 1–3.

### 4.3. Histological Analysis and Immunostaining

Testicular tissues were immersed in 4% paraformaldehyde and fixed overnight at 4 °C. Afterwards, the samples were dehydrated stepwise with ethanol (70% to 100%), treated with xylene, embedded in paraffin, and sectioned at 5 μm thickness using a Leica microtome (GmBH; Nussloch, Germany). Hematoxylin and eosin (H&E) staining was performed on paraffin sections. A blinded analyst evaluated five distinct regions of each testicular sample under a light microscope to ensure objectivity. Immunohistochemistry was performed as described by Lee et al., 2024 [45]. Stained tissues were examined using an Olympus IX73 microscope (Tokyo, Japan). Three biological replicates were analyzed per group unless otherwise indicated. In each replicate, at least 40 seminiferous tubules from four testicular cross-sections were analyzed. Primary antibodies used are listed in Table S1.

For immunocytochemistry, cells were cultured on chambered cover glasses and treated with 100 μM HEX for 24 h, following which the cover glasses were rinsed with PBS and immediately fixed with 4% formaldehyde for 15 min at room temperature (24 °C). Next, the cells were washed and incubated with primary antibodies diluted in a blocking buffer (PBS containing 0.05% Triton X-100 and 1% bovine serum albumin) for 24 h at 4 °C. Following thorough PBS washes, the cells were incubated with secondary antibodies for 1 h at 24 °C. After staining, the samples were mounted using DAPI (Vector Laboratories Inc., Burlingame, CA, USA), and fluorescence images were acquired using an Olympus IX73 microscope. Table S1 provides the detailed antibody information.

### 4.4. Western Blotting

Proteins were extracted using RIPA buffer (Thermo Fisher Scientific, Rockford, IL, USA) supplemented with protease inhibitors (Roche Diagnostics, Mannheim, Germany). Equal amounts of protein (30 μg) were separated by SDS-PAGE on 4–20% gels (Bio-Rad; Hercules, CA, USA; #456–1096) and transferred onto polyvinylidene fluoride membranes. The membranes were exposed to primary antibodies at 4 °C for 20 h (details in Table S1), followed by incubation with secondary antibodies for 1 h at room temperature (24 °C). Protein signals were visualized with the Western Pico chemiluminescent substrate (Thermo Scientific; Rockford, IL, USA) and recorded using the Bright™ Imaging System (Thermo Fisher Scientific; Waltham, MA, USA). To ensure equal loading, B-actin (ACTB) was used as a loading control. The primary antibodies used are listed in Table S1.

### 4.5. Quantitative PCR Analysis

Total RNA was isolated using the RNeasy Mini Kit (Qiagen; Hilden, Germany), and residual genomic DNA was removed using on-column DNase digestion (Qiagen). The resulting RNA was reverse transcribed into complementary DNA (cDNA) using the RevertAid First Strand cDNA Synthesis Kit (Thermo Scientific; Rockford, IL, USA). For transcript quantification, a qPCR was conducted using a QuantStudio™ 1 Real-Time PCR System (Applied Biosystems; Foster City, CA, USA) with SYBR Green Master Mix (Thermo Fisher Scientific; Waltham, MA, USA). Details of the cycling conditions and analytical procedures have been described previously [46]. Gene expression values were normalized to GAPDH and expressed as log2-transformed data. Primer sequences, including those designed using Primer3, are listed in Table S2.

### 4.6. Testosterone Measurement

Cardiac puncture was performed to obtain blood samples from which serum was isolated. Serum testosterone concentrations were quantified using a mouse-specific ELISA kit (Cusabio Biotech; Houston, TX, USA) following the manufacturer’s instructions.

### 4.7. Cell Staining with CellROX, MitoSOX, and BODIPY

Leydig cells (1 × 10^5^) were seeded per well on a cover glass in a 6-well plate (two passages). After 24 h for stabilization, 100 μM HEX was added to the culture medium for an additional 24 h. The cells were stained with CellROX (c10444; Invitrogen, Carlsbad, CA, USA), MitoSOX (M36008; Carlsbad, CA, USA), and BODIPY (HY-W090090; MedChemExpress, Monmouth Junction, NJ, USA) following the manufacturer’s instructions. Images were obtained using a fluorescence microscope (IX73; Olympus, Tokyo, Japan).

### 4.8. Statistical Analysis

Data were expressed as mean ± standard deviation (SD). Statistical analysis was performed using IBM SPSS Statistics software (version 30.0; IBM Corp., Armonk, NY, USA). Differences between the control group and each HEX exposure group were evaluated using independent samples *t*-tests (two-sided tests). A *p*-value less than 0.05 was considered statistically significant, and significance levels were indicated as * *p* < 0.05, ** *p* < 0.01, and *** *p* < 0.001. All graphs were created using GraphPad Prism software (version 9.3; GraphPad Software, San Diego, CA, USA).

## 5. Conclusions

Our study provides novel evidence that HEX exposure induces male reproductive toxicity by disrupting the Leydig cell–spermatogenesis axis, potentially through oxidative stress-associated molecular alterations consistent with ferroptosis. Although no overt morphological abnormalities were observed, HEX increased oxidative stress predominantly within the testicular interstitial compartment, as demonstrated by enhanced DHE and HO-1 staining. Consistent with these in vivo findings, primary Leydig cells exposed to HEX exhibited molecular alterations associated with ferroptosis, including increased lipid peroxidation, reduced GPX4 expression, and impaired steroidogenic function. These changes were accompanied by decreased expression of 3β-HSD and Cyp11a1, reduced serum testosterone levels, and subsequent impairment of germ cell development, as evidenced by decreased DDX4 and SYCP3 expression together with reduced sperm motility.

Several limitations of this study should be acknowledged. First, the HEX dose used in this study was selected to investigate the underlying mechanisms of reproductive toxicity rather than to mimic environmentally relevant exposure levels. Therefore, our findings should be interpreted within the context of mechanistic toxicology and highlight early biological responses to HEX exposure. Second, although our data consistently demonstrated oxidative stress and dysregulation of multiple ferroptosis-associated molecular markers, definitive confirmation of ferroptosis will require future pharmacological rescue experiments using specific ferroptosis inhibitors, such as Ferrostatin-1 or Liproxstatin-1. Third, only a single in vivo dose of HEX was evaluated; therefore, additional dose–response studies are warranted to further define the toxicological relevance of HEX exposure. Finally, although primary Leydig cells with high purity were successfully isolated, the low-level expression of Sox9 suggests minimal Sertoli cell contamination, which should be considered when interpreting the in vitro findings. In conclusion, HEX exposure disrupts testicular function by inducing oxidative stress and impairing Leydig cell steroidogenesis, accompanied by molecular alterations consistent with ferroptosis. These events may contribute to defective spermatogenesis and male reproductive dysfunction despite the absence of overt structural abnormalities. Collectively, our findings provide mechanistic insight into the early reproductive toxicity of HEX and suggest that Leydig cell dysfunction and steroidogenic signaling represent early pathological events in HEX-induced male reproductive toxicity.

## Data Availability

The original contributions presented in this study are included in the article/Supplementary Material. Further inquiries can be directed to the corresponding author.

## Supplementary Materials

The following supporting information can be downloaded at: https://www.mdpi.com/article/10.3390/ijms27146428/s1.
