# Supplementary material for: Hexaconazole-Induced Male Reproductive Toxicity Through ROS-Mediated Ferroptosis and Impaired Leydig Cell Steroidogenesis
Source: Int J Mol Sci. 2026 Jul 20;27(14):6428. doi: 10.3390/ijms27146428 (PMC13409936; doi:10.3390/ijms27146428)
Supplement: Supplementary file 1 [file ijms-27-06428-s001.zip › ijms-4362946-supplementary.pdf]

Table S1. Primary antibodies used for immunostaining and western blotting (WB).

Antibody	Company	Catalog N umber	Diluted
DDX4 (IHC), (WB)	Abcam	ab13840	1:100, 1:1000
SYCP3 (IHC), (WB)	Abcam	ab97672	1:100, 1:1000
3β-HSD (IHC), (WB)	Santa Cruz Biotech	Sc-30820	1:100, 1:1000
HO-1(IHC), (WB)	Santa Cruz Biotech	Sc-136960	1:100, 1000
KEAP1 (WB)	Santa Cruz Biotech	Sc-514914	1:1000
NRF2 (WB)	Santa Cruz Biotech	Sc-365949	1:1000
GPX4 (IHC), (WB)	Santa Cruz Biotech	Sc-166570	1:100, 1:1000
FTH1(WB)	Cell Signaling	#4393S	1:1000
xCT/SLC7A11 (WB)	Cell Signaling	#12691s	1:1000
ACTB (WB)	Santa Cruz Biotech	SC-47778	1:100

Table S2. Primers used for reverse transcription-polymerase chain reaction using mouse cDNA.

**Gene** (NCBI Gene Accession No.)	**Forward primer**	**Reverse primer**
Gapdh (XM_032905639.1)	5′- GTCGGTGTGAACGGATTTG-3′	5′-CTTGCCGTGGGTAGAGTCAT-3′
Ddx4 (NM_001145885)	5′-CCGCATGGCTAGAAGAGATT-3′	5′-TTCCTCGTGTCAACAGATGC-3′
Sycp3 (NM_011517)	5′-CAGATGCTTCGAGGGTGTG-3′	5′-AAGGTGGCTTCCCAGATTTC-3′
Piwil2 (AF285586.1)	5′-TGGTGATTGGAATGGATGTG-3′	5′-ATGTGGCATCTGGAACACC-3′
Sod1 (AH002084)	5′- GGGTTCCACGTCCATCAGTA -3′	5′-AGTCACATTGCCCAGGTCTC -3′
Cat (NM_009804)	5′- GCAGATACCTGTGAACTGTC-3	5′- GTAGAATGTCCGCACCTGAG-3
Gpx1 (NM_008160.6)	5′- TTCGGACACCAGGAGAATGG-3′	5′- TAAAGAGCGGGTGAGCCTTC-3′
Ho-1 (NM_010442.2)	5′- AACAAGCAGAACCCAGTCTAT-3′	5′- AGGTAGCGGGTATATGCGTGGGC-3′
Nrf2 (U20532.1)	5′- TCTCCTCGCTGGAAAAAGAA-3′	5′- AATGTGCTGGCTGTGCTTTA-3′
Nqo1 (NM_008706.5)	5′- CTTTAGGGTCGTCTTGGC-3′	5′- CAATCAGGGCTCTTCTCG-3′
Tfrc (NM_011638.4)	5′- GAGTGGCTACCTGGGCTAT-3′	5′- TGTCTGTCTCCTCCGTTT-3′
Slc11a2 (NM_001356952.1)	5′- GAGCCCTTCACCACCTAC-3′	5′- AACGCCCAGAGTTTACGA-3′
Ncoa4 (NM_019744)	5′- CGCCAGACCATCACCACA-3′	5′- GTGCCACTGGATGCTGACTT-3′
Acsl4 AB033885.1)	5′- ACTTTCCACTTGTGACTTTAT-3′	5′- CTTCAGTTTGCTTTCCAG-3′
Fth1 (NM_010239))	5′- ACCTGGAGTTGTATGCCT-3′	5′- TGAGATTGGTGGAGAAAGT-3′
Gpx4 (NM_008162)	5′- ATTCTCAGCCAAGGACAT-3′	5′- CAGGATTCGTAAACCACA-3′
Bax (NM_007527)	5′-GCTGACATGTTTGCTGATGG-3′	5′-GATCAGCTCGGGCACTTTAG-3′
Bad (NM_007522)	5′-GCCCTAGGCTTGAGGAAGTC-3′	5′-GGCTCAAACTCTGGGATCTG-3′
Bcl2 (NM_007523)	5′-GGGATGCCTTTGTGGAACTA-3′	5′-CTCACTTGTGGCCCAGGTAT-3′
Sox9 (NM_011448)	5 ′ - AGTACCCGCATCTGCACAA -3′	5′-TACTTGTAATCGGGGTGGTC- 3 ′
Cyp11al (NM_019779.4)	5′- GACAATGGTTGGCTAAACCTG -3′	5′- GGGTCCACGATGTAAACTGAC-3′
Insl3 (NM_013564)	5′-TGCAGTGGCTAGAGCAGAGAC-3′	5′-GAGAAGCCTGGAGAGGAAGC-3′
Cyp17a1 (NM_007809)	5′-TCCAGCATTGGAGAGTTTGC-3′	5′-ATGAGATGGCTTCCTGTTGG-3′
3β-HSD1(NM_001304800)	5′-AATCTGAAAGGTACCCAGAA-3′	5′-TCATCATAGCTTTGGTGAGG-3′
17β-HSD3 (NM_008291)	5′- GCTCAACGATTCCTCCTGAC-3′	5′-CCACCCAACCCTAACTCTACC-3′
